# Brine Shrimp Cytotoxicity, Anti-inflammatory and Analgesic Properties of *Woodfordia fruticosa* Kurz Flowers

**Published:** 2012

**Authors:** Yogesh Baravalia, Yogeshkumar Vaghasiya, Sumitra Chanda

**Affiliations:** Phytochemical, Pharmacological and Microbiological Laboratory, Department of Biosciences, Saurashtra University, Rajkot-360 005, Gujarat, India.

**Keywords:** *Woodfordia fruticosa*, Cytotoxicity, Anti-inflammatory, Carrageenan, Histamine, Dextran, Cotton pellet

## Abstract

The present study was designed to assess the cytotoxicity, anti-inflammatory and analgesic properties of methanol extract of *Woodfordia fruticosa *flowers. Cytotoxic activity of methanol extract of *Woodfordia fruticosa *flowers was tested using *Artemia salina *(Brine shrimp) bioassay. Two doses (400 and 600 mg/Kg) were evaluated for the anti-inflammatory activity against the carrageenan, histamine, dextran, serotonin and formaldehyde-induced rat paw edema, cotton pellet-induced granuloma and formaldehyde-induced analgesia in rats. In cytotoxicity study, extract caused 73% mortality of Brine shrimp larvae after 24 h at a concentration of 1000 μg/mL. The results of the anti-inflammatory study showed that the extract produced significant (p < 0.05) decrease in paw volume in different models of paw edema. The extract also inhibited the formation of granuloma in cotton pellet-induced granuloma and reduced the frequency of formaldehyde-induced paw licking. These results showed that the methanol extract of *Woodfordia fruticosa *flowers have weak cytotoxic and potent anti-inflammatory compounds and justifies the traditional uses for the treatment of inflammatory conditions.

## Introduction

The use of medicinal plants in curing diseases is as old as human being. The World Health Organization (WHO) has long recognized and drawn the attention of many countries to the ever increasing interest of the public in the use of medicinal plants and their products in the treatment of various ailments. These plants which are found in our environment enjoy wide acceptability through the population and serve as cheaper alternatives to orthodox medicine (1). However, many plants are known to be toxic. For this reason, the present research is carried out in order to determine the pharmacological action and the toxicity of medicinal plants (2). The brine shrimp cytotoxicity assay was considered as a convenient probe for preliminary assessment of toxicity (3).

Inflammation is an important physiological reaction which occurs in response to a wide variety of injurious agents (*e.g*. bacterial infection, physical trauma, chemicals or any other phenomenon) ultimately aiming to perform the dual function of limiting damage and promoting tissue repair (4). However, excessive or persistent inflammation causes a variety of pathological conditions, such as bacterial septic shock and rheumatoid arthritis (5). It is necessary to manage the hyper-inflammation to a useful level, to change the clinical manifestation of the disease. The scientific interest has now been diverted towards the natural compounds which are biocompatible, safe and also cost-effective. Thus, efforts are continuously being made to identify such agents and to validate their scientific authenticity (6).

*Woodfordia fruticosa *Kurz (syn. *Woodfordia floribunda *Salisb.) belongs to the family Lythraceae and is locally known as Dhavdi (Gujarat, India) (7). All parts of this plant possess valuable medicinal properties viz. anti-inflammatory, anti-tumor, hepatoprotective and free radical scavenging activity (8-11), but its flowers are in maximum demand (12). The flowers are being used in the preparation of Ayurvedic fermented drugs called Aristhas and Asavas, and are very popular in the Indian subcontinent as in other South Asian countries (13). In the current study, the aim was to study the brine shrimp cytotoxicity, anti-inflammatory (acute and chronic) and analgesic activity of the methanol extract of *Woodfordia fruticosa *Kurz. flowers.

## Experimental

*Plant material*

The fresh flowers of *Woodfordia fruticosa *were collected from Junagadh (Girnar region), Gujarat, India, in March 2008. The plant was compared with voucher specimen (voucher specimen No. PSN303) deposited by Dr. P.S. Nagar, at Department of Biosciences, Saurashtra University, Rajkot, Gujarat, India.

*Preparation of the extract*

The flowers were washed under tap water, air dried, homogenized to fine powder and stored in airtight bottles. Ten grams of dried powder was first defatted with petroleum ether and then extracted with methanol using Soxhlet apparatus (14). The solvent was evaporated to dryness and the dried crude extract was stored in airtight bottle at 4°C. The percentage yield of methanol extract was 36%. For testing, the *Woodfordia fruticosa *methanol (WFM) extract was dissolved in sterile distilled water and diluted to the desired concentrations.

*Chemicals*

All chemicals were of analytical-reagent 852

grade and obtained from the following sources: Carrageenan and dextran from Hi media (Mumbai, India); histamine, serotonin and diclofenac sodium from Sigma Aldrich (St. Louis, MO, USA); petroleum ether, methanol from Merck (Darmstadt, Germany) and biochemical kits were purchased from Span Diagnostics Ltd (Sachin, Surat, India).

*Test sample preparation for Brine shrimp bioassay*

Test sample was dissolved in DMSO (Dimethyl sulfoxide) to obtain stock solution of 1 mg/mL concentration. The final concentration of DMSO in the assay volume was kept at 2% to prevent possible false effects originating from DMSO toxicity. Pure DMSO and artificial seawater were used as negative control and potassium dichromate was used as the reference standard for the cytotoxicity assay.

*Hatching of Brine shrimp cysts*

Fresh cysts were produced from Fisheries Research Center, Okha, Gujarat, India. The cysts were hatched in a hatching tank containing artificial seawater made through dissolving a commercial marine salt (2%) in RO water (mineral water). The tank was well aerated with the aid of an air pump and the proper light source (1000-4000 lux) was also provided. The nauplii were hatched within 24-36 h at 30-35°C.

*Brine shrimp lethality test*

The toxicity of compound was tested at various concentrations viz. 10, 100, 250, 500, 750 and 1000 μg/mL in seawater containing 2% DMSO (v/v). Ten nauplii were used in each test. Three replications were used for each concentration. A parallel series of tests with the standard potassium dichromate solution (LC_50_ = 20-40 μg) were tested and the blank control was always included. After 24 h, survivors were counted using a dissection microscope and the percentage of the mortality (%M) of each dose was calculated as compared with control. Cytotoxicity was considered significant if the LC_50_-value was less than 20-30 μg/mL (15).

*Animals*

Wistar albino rats of either sex (180-220 g) were used for the study. The animals were obtained from Sarabhai Research Center (SRC), Baroda. All the rats were kept in standard plastic rat cages with stainless steel coverlids and the wheat straw was used as a bedding material. The animals were kept at the animal house of Department of Biosciences, Saurashtra University, Rajkot. The animals were facilitated with standard environmental condition of photoperiod (12:12 h dark: light cycle) and temperature (25 ± 2°C). They were provided with commercial rat and mice feed (Pranav Agro Industries Ltd., Amruth Brand rat and mice pellet feed) and water was given *ad libitum*. The use of these animals and the study protocols were approved by CPCSEA recognized local ethical committee.

*Anti-inflammatory studies*

The animals were divided into four groups (six animals in each group) for anti-inflammatory studies. The dose of the *Woodfordia fruticosa *flowers was selected on the basis of therapeutic dose used in the traditional system of Ayurveda and the dose of Diclofenac was selected on the basis of literature survey as follows: Group I: Vehicle-treated control (distilled water), Group II: Methanol extract of *Woodfordia fruticosa *(400 mg/Kg body weight (WFM-400)), Group III: Methanol extract of *Woodfordia fruticosa *(600 mg/Kg body weight (WFM-600)), Group IV: Diclofenac sodium (- 10 mg/Kg body weight (diclofenac - 10)).

*Carrageenan-induced rat paw edema*

Carrageenan-induced rat paw edema was done through the method of Winter *et al. *(16). The inflammation was induced through injecting 0.1 mL of freshly prepared carrageenan (1%) aqueous suspension in normal saline underneath the plantar tissue of the right hind paw of the rats. The different groups of rats were administered with WFM (400 and 600 mg/Kg, p.o.) and diclofenac (10 mg/Kg, p.o.). The control group received vehicle (distilled water, 10 mL/Kg, p.o.). 1 h after the drug treatment and the paw edema was induced through the injection of carrageenan (an edematogenic agent). The paw volume was measured using a Plethysmometer. The measures were determined at 0 h (Vo: before the edematogenic agent injection) and 1, 2, 3, 4 and 5 h later (Vt). The difference between Vt and Vo was taken as the edema value. The percentage of inhibition was calculated according to the following formula:

$\mathrm{\% inhibition}=\frac{(\mathrm{Vt}-\mathrm{Vo})\mathrm{control}-(\mathrm{Vt}-\mathrm{Vo})\mathrm{treated}}{(\mathrm{Vt}-\mathrm{Vo})\mathrm{control}}\times 100$

*Histamine, dextran and serotonin-induced rat paw edema*

The animals were treated in a manner similar to that of carrageenan-induced paw edema model. A volume of 0.1 mL freshly prepared of 1% histamine, dextran and serotonin (17-19) were injected in the paw of rats. The paw volume was measured as mentioned in the carrageenan-induced paw edema model.

*Formaldehyde-induced rat paw edema*

The inflammation was induced through the injection of 0.1 mL of freshly prepared Formaldehyde (3%) underneath the plantar tissue of the right hind paw of rats (20). The test drug was administered consecutively for seven days to all the groups. On seventh day, after 1 h of drug administration, the paw edema of the rat was induced by subplantar injection of formaldehyde solution. The paw volume was determined at 0 h and at 3, 24 and 48 h after the formaldehyde injection as described in the carrageenan model.

*Cotton pellet-induced granuloma in rats*

The effect of WFM on the chronic phases of inflammation was assessed in the cotton pellet-induced granuloma rat model, as described by Swingle and Shideman (21). Autoclaved cotton pellets weighing 100 mg each, were implanted subcutaneously, one on each side of the abdomen of the animal, through a small ventral incision of rats anesthetized with ether. The different groups of rats were administered with WFM (400 and 600 mg/Kg, p.o.) and diclofenac (10 mg/Kg. p.o.) once daily for 7 consecutive days from the day of cotton pellet insertion. The control group received vehicle (distilled water, 10 mL/Kg, p.o.). On the 8^th^ day, the animals were sacrificed and the cotton pellets were removed, dried at 60°C for 24 h and their mass was determined. The results are expressed as mg granulation tissue formed per 100 g body weight.

*Biochemical analysis*

On the 8^th^ day, the animals were sacrificed under mild ether anesthesia and the blood was collected in clean centrifuge tubes for biochemical estimations. The serum was obtained by centrifugation and various serum biochemical parameters, viz. total protein (22), albumin (23), acid phosphatase (24) and alkaline phosphatase (25) were estimated using Span Diagnostics test kits. The absorbance of all the biochemical parameters was measured in a UV-VIS Spectrophotometer (Shimadzu, Tokyo, Japan).

*Analgesic study*

*Formaldehyde-induced paw licking response in rats*

The effect of WFM on formaldehyde-induced paw licking response was evaluated through the procedure of Magali *et al*. (26). The test drug was administered once daily for seven consecutive days to all the groups. On seventh day, after 1 h of drug administration, the subplantar injection of 0.1 mL of 3% formaldehyde solution in normal saline was injected. After the injection of formaldehyde, the animals were kept under observation for 30 min. The amount of time spent licking the injected paw was noted and considered to be indicative of pain. The time taken for the onset of paw licking was initially measured. The first nociceptive responses normally peaked 5 min after the formaldehyde injection and the second phase was 15-30 min after the formaldehyde injection, representing the neurogenic and inflammatory pain. Therefore, the frequency of paw licking was measured in five intervals at 0-5 min., 6-10 min., 11-15 min., 16-20 min., and 21-30 min.

*Statistical analysis*

In this study, recorded values are expressed as mean ± standard error of mean (SEM). Statistical significance was determined using one-way ANOVA followed by Student’s t-test. Values are considered statistically significant at p < 0.05 for t-test and f < 0.05 for ANOVA. In cytotoxicity study, LC_50_-values and 95% of confidence intervals were determined.

## Results

*Brine shrimp cytotoxicity*

Brine shrimp lethality activity of the WFM is shown in Table 1. The crude extract showed 73% mortality at 1000 μg/mL concentration and its LC_50_-value was 763.34 μg/mL which was considered moderately toxic. Reference standard potassium dichromate showed LC_50_-value (38 μg/mL). No mortality was found in negative control (DMSO) group.

**Table 1:** Result of Brine shrimp lethality bioassay

**Test material**	**% Mortality under the concentration studied (μg/mL)**						**LC**_50_ **(μg/mL)**	**95% Confidence interval**	**Toxicity profile**
**(w/v)**	**10**	**100**	**250**	**500**	**750**	**1000**			
*W. fruticosa *methanol extract	0	3.33 ± 0.32	6.67 ± 0.32	33.33 ± 0.32	43.33 ± 0.32	73.33 ± 0.32	763.34	675.65-877.09	Toxic
**(w/v)**	**10**	**20**	**25**		**30**	**50**			
K_2_Cr_2_O_7_	1.33 ± 0.20	8.00 ± 0.49	22.67 ± 0.80		46.00 ± 0.94	70.00 ± 0.65	38.18	36.23 – 40.44	Toxic
**(v/v)**	**0.1%**	**0.5%**		**1%**	**2.5%**				
DMSO	0	0		0	0		0	0	Non-toxic

Values are the mean of triplicate studies (mean ± SEM), N = 10 (no. of shrimps). Score for LC_50_: Highly-toxic < 20 μg/mL, Toxic-upto 1000 μg/mL, Non-toxic > 1000 μg/mL

*Carrageenan-induced rat paw edema*

The results of anti-inflammatory activity of WFM on carrageenan-induced paw edema are shown in Table 2. The lower dose, *i.e*. WFM-400, showed inhibition at both early and late phase; though maximum inhibition was at late phase (69%, p < 0.01). The higher dose, *i.e*. WFM-600, also showed maximum anti-inflammatory activity at late phase (42%) but this activity was less than that of WFM-400 at both early and late phase. The standard Diclofenac-10 showed maximum activity at early phase (59%, p < 0.01). In this model, the lower dose showed more inhibition of edema formation than standard Diclofenac.

**Table 2:** Anti-inflammatory activity of methanol extract of *Woodfordia fruticosa *flowers in carrageenan-induced rat paw edema

**Treatment ** **(mg/K**g)	**%Increase in paw volume**									
	**After 1 h**		**After 2 h**		**After 3 h**		**After 4 h**		**After 5 h**	
	**Vol. Increase**	**%Change**	**Vol. Increase**	**%Change**	**Vol. Increase**	**%Change**	**Vol. Increase**	**%Change**	**Vol. Increase**	**%Change**
Control	16.49 ± 3.12		27.49 ± 3.49			34.37 ± 4.45			33.23 ± 5.64	32.08 ± 5.16
WFM-400	8.05 ± 1.31^*^	51.16↓	12.06 ± 2.25^**^	56.11↓	15.16 ± 3.09^**^	55.90↓	12.87 ± 2.26^**^	61.26↓	9.93 ± 2.85^**^	69.04↓
WFM-600	10.76 ± 2.98	34.78↓	20.03 ± 3.46	27.15↓	25.22 ± 4.34	26.63↓	24.67 ± 4.72	25.77↓	18.46 ± 5.06	42.47↓
Diclofenac-10	9.00 ± 1.58	45.44↓	13.34 ± 3.86^*^	51.47↓	13.86 ± 2.33^**^	59.67↓	17.68 ± 3.55^*^	46.79↓	17.01 ± 4.39^*^	46.97↓
ANOVA-test		f < 0.05			f < 0.01		f < 0.05		f < 0.05	

Values are expressed as mean ± SEM, (n = 6). ^*^p < 0.05; ^**^p < 0.01

*Histamine-induced rat paw edema*

The results of anti-inflammatory activity of WFM on histamine-induced paw edema are shown in Table 3. In this model, the WFM at both dose levels and standard diclofenac showed anti-inflammatory activity at late phase. A clear dose-dependent inhibition of paw edema was observed. The percentage inhibition of WFM-400 was 36% (p < 0.05) while that of WFM-600 was 46% (p < 0.01). The later was nearer to standard Diclofenac-10 (55%, p < 0.01).

**Table 3:** Anti-inflammatory activity of methanol extract of *Woodfordia fruticosa *flowers in histamine-induced rat paw edema

**Treatment** **(mg/K**g)	**% Increase in paw volume**									
	**After 1 h**		**After 2 h**		**After 3 h**		**After 4 h**		**After 5 h**	
	**Vol. Increase**	**%Change**	**Vol. Increase**	**%Change**	**Vol. Increase**	**%Change**	**Vol. Increase**	**%Change**	**Vol. Increase**	**%Change**
Control	43.32 ± 3.93		48.81 ± 4.65			48.87 ± 5.75	47.32 ± 5.84		44.00 ± 4.38	
WFM-400	40.49 ± 2.77	6.51↓	36.78 ± 2.45^*^	24.65↓	31.93 ± 1.90^*^	34.66↓	29.91 ± 1.98^*^	36.79↓	28.00 ± 2.95^*^	36.35↓
WFM-600	37.13 ± 2.59	14.28↓	35.35 ± 4.46^*^	27.58↓	28.77 ± 3.71^*^	41.13↓	27.82 ± 3.58^*^	41.20↓	23.77 ± 3.45^**^	45.97↓
Diclofenac-10	30.31 ± 4.19^*^	30.02↓	28.38 ± 4.30^**^	41.86↓	27.46 ± 3.20^**^	43.82↓	21.74 ± 4.60^**^	54.05↓	19.83 ± 3.30^**^	54.93↓
ANOVA-test			f < 0.05		f < 0.01		f < 0.01		f < 0.001	

Values are expressed as mean ± SEM, (n = 6). ^*^p < 0.05; ^**^p < 0.01

*Dextran-induced rat paw edema*

The results of anti-inflammatory activity of WFM on dextran-induced paw edema are shown in Table 4. In this model, a clear dose-dependent inhibition of paw edema was observed at both early and late phases. The higher dose showed distinctly more inhibition than the lower one at both phases (p < 0.05). The anti-inflammatory activity of higher dose was more at later phase (50%) than the early one. The standard Diclofenac showed poor anti-inflammatory activity in this model.

**Table 4:** Anti-inflammatory activity of methanol extract of *Woodfordia fruticosa *flowers in dextran-induced rat paw edema

**Treatment ** **(mg/Kg)**	**%Increase in paw volume**									
	**After 1 h**		**After 2 h**		**After 3 h**		**After 4 h**		**After 5 h**	
	**Vol. Increase**	**%Change**	**Vol. Increase**	**%Change**	**Vol. Increase**	**%Change**	**Vol. Increase**	**%Change**	**Vol. Increase**	**%Change**
Control	44.80 ± 4.85		47.10 ± 6.08		41.90 ± 4.45		33.77 ± 5.82		25.43 ± 3.88	
WFM-400	33.71 ± 3.41	24.77↓	36.49 ± 3.56	22.53↓	30.07 ± 5.91	28.24↓	22.45 ± 3.01	33.53↓	20.75 ± 3.17	18.43↓
WFM-600	27.51 ± 3.66^*^	38.58↓	27.61 ± 4.18^*^	41.39↓	26.96 ± 1.59^*^	35.66↓	16.82 ± 3.09^*^	50.18↓	12.85 ± 3.23^*^	49.46↓
Diclofenac-10	42.21 ± 4.82	5.78↓	40.05 ± 4.60	14.98↓	36.41 ± 4.69	13.10↓	26.62 ± 4.68	21.17↓	23.91 ± 3.52	6.00↓
ANOVA-test	f < 0.05									

Values are expressed as mean ± SEM, (n = 6). ^*^p < 0.05; ^**^p < 0.01

*Serotonin-induced rat paw edema*

The results of anti-inflammatory activity of WFM on serotonin-induced paw edema are shown in Table 5.

**Table 5:** Anti-inflammatory activity of methanol extract of *Woodfordia fruticosa *flowers in serotonin-induced rat paw edema

**Treatment ** **(mg/Kg)**	**% Increase in paw volume**									
	**After 1 h**		**After 2 h**		**After 3 h**		**After 4 h**		**After 5 h**	
	**Vol. Increase**	**%Change**	**Vol. Increase**	**%Change**	**Vol. Increase**	**%Change**	**Vol. Increase**	**%Change**	**Vol. Increase**	**%Change**
Control	17.46 ± 2.88		22.71 ± 4.21		34.79 ± 5.45		27.50 ± 3.55		21.03 ± 3.57	
WFM-400	9.35 ± 1.05^*^	46.44↓	13.43 ± 2.79	40.88↓	18.31 ± 2.33^*^	47.37↓	14.77 ± 2.99^*^	46.30↓	4.79 ± 2.29^**^	77.22↓
WFM-600	6.89 ± 1.31^**^	60.52↓	14.52 ± 1.58	36.04↓	17.53 ± 3.71^*^	49.61↓	13.62 ± 4.56^*^	50.47↓	7.91 ± 2.00^**^	62.39↓
Diclofenac-10	6.45 ± 3.25^*^	63.05↓	8.53 ± 2.78^*^	62.45↓	10.82 ± 3.74^**^	68.91↓	6.95 ± 3.78^**^	74.73↓	4.81 ± 2.37^**^	77.13↓
ANOVA-test	f < 0.05		f < 0.05		f < 0.01		f < 0.01		f < 0.001	

Values are expressed as mean ± SEM, (n = 6). ^*^p < 0.05; ^**^p < 0.01

The maximum paw thickness was observed at 3^rd^ h after the sub-planter injection in all groups. The animals treated with lower and higher doses of WFM (400 and 600 mg/Kg) produced statistically significant inhibition at 1^st^ (p < 0.05; p < 0.01, respectively), 2^nd^ and 3^rd^ (p < 0.05) and at 5^th^ h (p < 0.01). Standard drug Diclofenac-10 showed significant decrease in paw volume at 1^st^, 2^nd^ (p < 0.05) and at 3^rd^, 4^th^ and 5^th^ h (p < 0.01). The maximum decrease of paw volume in both doses of WFM and Diclofenac-treated groups was found at 5^th^ h (f < 0.001). The percent inhibition of WFM-400 group at 5^th^ h was the same as that of standard group. The extract effectively suppressed the inflammation produced by serotonin.

*Formaldehyde-induced rat paw edema*

The results of anti-inflammatory activity of WFM in formaldehyde-induced paw edema are shown in Table 6. The subcutaneously injection of formaldehyde into the hind paw of rats produces localized inflammation. The administration of WFM-400, WFM-600 and Diclofenac-10 daily for 7 days successfully significantly (f < 0.001) inhibited edema induced by formaldehyde. WFM-400 and WFM-600 group showed maximum decrease in paw volume at 3 h (p < 0.05 and p < 0.01 respectively). Diclofenac-10 group showed decrease in paw volume at 3 h (45.65%, p < 0.001) and the decrease in paw volume at 48 h was almost the same (46.06%, p < 0.01).

**Table 6:** Anti-inflammatory activity of methanol extract of *Woodfordia fruticosa *flowers in formaldehyde-induced rat paw edema

**Treatment (mg/Kg)**	**%Increase of in paw volume**					
	**After 3 h**		**After 24 h**		**After 48 h**	
	**Vol. Increase**	**%Change**	**Vol. Increase**	**%Change**	**Vol. Increase**	**%Change**
Control				57.94 ± 1.97	43.11 ± 2.23	
FMM-400	34.23 ± 5.23^*^	36.53	36.96 ± 2.89^**^	36.22	32.26 ± 5.13	25.16
WFM-600	33.44 ± 3.87^**^	38.01	37.52 ± 6.64^*^	35.24	30.31 ± 4.13^*^	29.69
Diclofenac-10	29.32 ± 1.53^***^	45.65	33.11 ± 3.39^**^	42.86	23.26 ± 1.95^**^	46.06
ANOVA-test	f < 0.001		f < 0.01		f < 0.01	

Values are expressed as mean ± SEM, (n = 6). ^*^p < 0.05; ^**^p < 0.01; ^***^p < 0.001

*Cotton pellet-induced granuloma in rats*

The results of anti-inflammatory activity of WFM in cotton pellet-induced granuloma are shown in Table 7. WFM-400 and WFM-600 groups showed 1.28% and 11.45% decrease in granuloma formation respectively as compared with the control group, while standard diclofenac-10 group showed significant decrease in granuloma formation (37.15%, p < 0.001).

**Table 7:** Anti-inflammatory activity of methanol extract of *Woodfordia fruticosa *flowers in cotton pellet-induced granuloma in rats

**Treatment (mg/Kg)**	**Pellet weight (g/100 g body weight)**	**%Change**
Control	0.107 ± 0.005	-
WFM-400	0.106 ± 0.005	1.28↓
WFM-600	0.095 ± 0.005	11.45↓
Diclofenac-10	0.067 ± 0.004^***^	37.15↓
ANOVA-test	f < 0.001	

Values are expressed as mean ± SEM, (n = 6). ^***^p < 0.001

The results of changes in serum total protein and albumin levels in cotton pellet-induced granuloma are given in Table 8. 

**Table 8:** Effect of methanol extract of *Woodfordia fruticosa *flowers on serum biochemical parameters in cotton pellet-induced granuloma in rats

**Treatment (mg/Kg)**	**Total protein (g/dL)**	**Albumin (g/dL)**	**ACP (KA units)**	**ALP (KA units)**
Control	6.23 ± 0.063	3.56 ± 0.047	13.03 ± 1.09	52.07 ± 7.12
WFM-400	6.15 ± 0.078	3.36 ± 0.039	13.26 ± 1.22	36.97 ± 6.26
WFM-600	6.54 ± 0.063^**^	3.57 ± 0.089	13.13 ± 0.619	43.69 ± 5.04
Diclofenac-10	6.35 ± 0.136	3.32 ± 0.361	18.41 ± 5.43	60.25 ± 13.08

Values are expressed as mean ± SEM, (n = 6). ^*^p < 0.05; ^**^p < 0.01

The total protein level were increased at higher concentration (p < 0.01) and decreased at lower concentration of WFM, while Diclofenac-10 group showed increase in total protein level as compared to the control group. In standard and WFM-400 group, the albumin level was decreased as compared to the control group. The results of changes in serum ACP and ALP levels in cotton pellet-induced granuloma are given in Table 8. The ACP level in both doses of WFM was almost similar to that of control group. In standard group, the level of ACP was more as compared to the control group. The ALP levels were decreased in both the studied concentrations, and the decrease in lower concentration was more than that of the 

higher one. In contrast, the ALP level in the standard group was increased.

*Formaldehyde-induced paw licking test in rats*

The result of formaldehyde-induced paw licking test in rats of WFM is given in Table 9. The onset time of paw licking was measured after the formaldehyde injection. WFM-400 and WFM-600 showed 82.72% (p < 0.05) and 45.06% increase in onset time respectively, while diclofenac-10 group showed 26.54% increase in onset time as compared to the control group. After the formaldehyde injection, the frequency of paw licking was measured between 0-5 min, 6-10 min, 11-15 min, 16-20 min and 21-30 min. As compared to the control group, in the first phase (0-5 min), the extract reduced the paw licking up to 34.48% and 18.39% at WFM-400 and WFM-600 dose levels respectively, while Diclofenac-10 reduced the paw licking up to 21.84%. In second phase (16-20 min), both the extract and diclofenac reduced the dose-dependent paw licking up to 27.42%, 47.96% and 49.33 and (during 21-30 min) 24.04%, 29.23% and 35.07%, respectively.

**Table 9:** Analgesic activity of methanol extract of *Woodfordia fruticosa *flowers in formaldehyde-induced paw licking test in rats

**Treatment ** **(mg/Kg)**	**Onset time (Sec)**	**%Change**	**0-5 min**		**6-10 min**		**11-15 min**		**16-20 min**		**21-30 min**	
			**F**	**%Change**	**F**	**%Change**	**F**	**%Change**	**F**	**%Change**	**F**	**%Change**
Control	27.00 ± 6.04		14.50 ± 3.79		2.67 ± 0.76		5.33 ± 1.86		12.17 ± 2.57		25.67 ± 4.59	
WFM-400	49.33 ± 4.11^*^	82.72↑	9.50 ± 0.72	34.48↓	2.00 ± 1.44^*^	25.09↓	1.33 ± 0.71	74.98↓	8.83 ± 2.15	27.42↓	19.50 ± 4.52	24.04↓
WFM-600	39.17 ± 6.81	45.06↑	11.83 ± 1.76	18.39↓	0.50 ± 0.22	81.27↓	1.50 ± 0.72	71.86↓	6.33 ± 1.69	47.96↓	18.17 ± 5.34	29.23↓
Diclofenac-10	34.17 ± 4.43	26.54↑	11.33 ± 1.76	21.84↓	0.00 ± 0.00^*^	100.00↓	1.67 ± 0.61	68.73↓	6.17 ± 1.47	49.33↓	16.67 ± 2.19	35.07↓

Values are expressed as mean ± SEM, (n = 6). p ≤ 0.05*, F = Frequency, ↑: Increase, ↓: decrease

## Discussion

*Brine shrimp cytotoxicity*

The brine shrimp test represents a rapid, inexpensive and simple bioassay for testing the plant extract lethality which in most cases correlates reasonably well with cytotoxic and anti-tumor properties. Most often, a desired biological response is not due to one component but rather due to a mixture of bioactive plant components. Therefore, crude extracts must be screened for biological activity. The brine shrimp lethality assay has been proved to be a convenient system for monitoring biological activities of natural products (27). The degree of lethality was directly proportional to the concentration of the extract with LC_50 _763.34 μg/mL. The cytotoxic property of plant extract may be due to the presence of antitumor compounds in *Woodfordia fruticosa *flowers as reported by Yoshida *et al *(9).

*Anti-inflammatory study*

*Carrageenan-induced rat paw edema*

Carrageenan-induced rat paw edema is a widely used *in-vivo *acute model to predict the value of anti-inflammatory agents, which act through inhibiting the mediators of acute inflammation (28). Carrageenan-induced hind paw edema in rat is a biphasic event. The early phase (90-180 min) of the inflammation is due to the release of histamine, serotonin and similar substances and the later phase (270-360 min) is associated with the activation of kinin-like substances, *i.e.*, prostaglandins, proteases and lysosome (29). WFM inhibited the carrageenan-induced rat paw edema formation, at both early and later phases. This result tends to suggest that the inhibitory effect of the extract on edema formation is probably due to the inhibition of synthesis and/or the release of inflammatory mediators, especially the cyclooxygenase products. The carrageenan-induced paw edema test is effectively controlled with the arachidonate cyclooxygenase (COX) inhibitors due to its COX-dependent mechanism, thus, it is suggested that the WFM may possess arachidonate COX inhibitory property.

*Histamine-induced rat paw edema*

Histamine is another pro-inflammatory mediator involved in exudation and cell chemotaxis (30). The histamine is a basic amine related to the inflammatory and allergic process causing, among several effects, both vasodilatation and increase of vascular permeability (31). Edema was reduced through WFM in a dose-dependent manner till the end of 5^th^ h. The antihistaminic activity may be related to the inhibition of inflammation mediator formation. The extract may also inhibit the histamine release from mast cells and/or block histamine receptors.

*Dextran-induced rat paw edema*

Dextran is a polysaccharide of high molecular weight that induces the anaphylactic reaction after the injection in rats’ extremities, which is characterized through the extravasation and edema formation, as a consequence of liberation of histamine and serotonin from mast cells (32). In this study, WFM exhibited the dose-dependent inhibitory effect in dextran-induced paw edema and was capable to reduce the inflammation up to 5 h. The ability of the extract to reduce the edema volume suggests that the phytochemicals present in the extract may block or counteract the release of any mediators, alone or in combination.

*Serotonin-induced rat paw edema*

The extract effectively suppressed the inflammation produced by serotonin. WFM was able to significantly reduce paw edema, and these effects were similar to those exhibited through the group of rats treated with diclofenac. So, it may be suggested that its anti-inflammatory activity is possibly backed by its anti-serotonin activity.

*Formaldehyde-induced rat paw edema*

The inhibition of formaldehyde-induced paw edema in rats is one of the most widely used models to screen the anti-arthritic and anti-inflammatory agents as it closely resembles human arthritis (33). Subcutaneously injection of formaldehyde into hind paw of rats produces localized inflammation. In the present study, WFM significantly inhibited paw edema induced by formaldehyde. WFM showed significant decrease in paw volume till 48 h with both doses, which suggests its long duration of action.

*Cotton pellet-induced granuloma in rats*

Cotton pellet-induced granuloma formation is a typical feature of an established chronic inflammatory reaction and can serve as a chronic and subchronic inflammatory test model for the investigation of anti-arthritic substances (34). The extract showed decrease in granuloma formation. This reflects its efficacy to reduce an increase in the number of fibroblasts and synthesis of collagen with mucopolysaccharide, which are natural proliferative events of granulation tissue formation. At higher concentration of extract the protein level was increased. An increase in protein level at higher concentration of methanol extract of *Polyalthia longifolia *was also reported by Tanna *et al *(35). The rise in protein and albumin levels at high doses suggests stabilization of the endoplasmic reticulum, leading to protein synthesis (36).

*Formaldehyde-induced paw licking test in rats*

The formaldehyde test has been described as a convenient method for producing and quantifying pain in rats (37). The test employs an adequate painful stimulus to which the animals show a spontaneous response and it is sensitive to commonly used analgesics. The advantage of the formaldehyde model of nociception was that it could discriminate between the central and peripheral pain components. The test consists of two different phases which could be separated in time: the first one that occurs on the first 5 min after the formaldehyde injection was generated in the periphery through the activation of nociceptive neurons via direct action of formaldehyde and the second phase that occurs between the 15^th^ and 30^th^ min after the formaldehyde injection, occurred through the activation of the ventral horn neurons at the spinal cord level (38). In the present study, WFM orally administered 1 h before the formaldehyde injection, was capable of inhibiting the paw licking process when compared with the control group. It was observed that rats treated with WFM showed nociceptive reaction, which was dose-related and inhibited in second phase of formaldehyde test. Drugs that act primarily on the central nervous system inhibit both phases equally, while peripherally acting drugs only inhibit the second phase. The second phase is an inflammatory response with inflammatory pain that can be inhibited through the anti-inflammatory drugs (39). The experimental results show that WFM produced better inhibitory effect during the second phase of the formaldehyde test. This experimental evidence suggests that the analgesic effect produced by WFM was involved in its peripheral action.

## Conclusion

In conclusion, the cytotoxicity exhibited by the crude extract was promising and this indicates the presence of potent bioactive compounds. The anti-inflammatory activity and anti-nociceptive activity of WFM, justifies the traditional uses of *Woodfordia fruticosa *for the treatment of pain and inflammatory-related ailments. Previous studies have revealed that the NSAIDs were capable of inhibiting this test; therefore, the mechanism of the anti-inflammation of WFM may be similar to the mechanism exerted by the NSAIDs. Since prostaglandins are known to take part in the inflammatory and nociceptive processes, the anti-inflammatory and anti-nociceptive activities of the WFM could be due to the modulation of the COX or prostaglandins actions.
